# Simulated Placement and Rounds (SPaR): Improving Self-Reported Preparedness for Foundation Training through Ward-Round-Based Simulation

**DOI:** 10.7759/cureus.116452

**Published:** 2026-09-17

**Authors:** Imaan Hirji, Megan Pearson

**Affiliations:** 1 Medical Education and Simulation, Royal Surrey NHS Foundation Trust, Guildford, GBR

**Keywords:** constructivism, kolb’s experiential learning, medical student confidence, simulation in medical education, transition to residency, undergraduate clinical skills education, undergraduate medical training, ward round-based simulation

## Abstract

Introduction

Research and anecdotal evidence show that undergraduate medical students often feel underprepared to start as foundation doctors, particularly in practical skills. To address this, a simulation project was designed to emulate a medical ward round, incorporating key skills used daily by foundation doctors, including documentation, prioritizing tasks, prescribing, clinical reviews, and communication. The aim was to provide students with a realistic, hands-on ward round experience in a controlled environment.

Materials and methods

A three-station simulation was delivered, comprising a consultant-led round, a student-led consultation, and managing an unstable patient. Final-year medical students (n = 18) completed the simulation during their medical rotations. Pre- and post-session questionnaires collected both quantitative data using a Likert scale and qualitative data through short-answer questions. Wilcoxon signed-rank tests and thematic analysis were applied to evaluate changes in self-reported confidence and preparedness.

Results

Following the simulation, students’ reported confidence improved across all domains. Mean preparedness increased from 5.06 ± 1.54 to 8.28 ± 0.93, documentation confidence from 5.33 ± 1.37 to 8.28 ± 1.15, and confidence in managing an unstable patient from 3.39 ± 1.01 to 6.44 ± 1.26. These results show considerable improvement in learners' self-reported preparedness across all domains, with all improvements statistically significant by Wilcoxon signed-rank test (p < 0.001). Thematic analysis identified scenario realism, practical prescribing, and effective feedback as key strengths. Suggestions for improvement included additional scenarios, clearer pre-briefing, and further discharge summary practice.

Conclusions

This study shows that ward-round-based simulation can improve final-year medical students’ confidence and self-reported preparedness for ward rounds. Embedding similar simulations within undergraduate curricula may better support transition to clinical roles, enhance practical skill development, and improve readiness for real-world ward rounds across training sites.

## Introduction

The transition from medical school to starting work as a foundation doctor can be intimidating. Doctors often feel unprepared for this transition, especially regarding the practical skills the job requires [1]. Ward rounds are a cornerstone of medical practice, and foundation doctors are expected to conduct them independently or with a senior team member from day one. While students are encouraged to practice and get involved during their medical school placements, experience can still vary. Formal teaching on skills such as documentation, task prioritization, and prescribing is often limited. This can leave students feeling out of their depth and lacking in confidence when starting their new clinical role [2].

Simulation is becoming a widely used tool in medical education [3]. It is often useful for experiential learning in a psychologically safe setting and allows an opportunity for direct feedback following a role-play patient-clinician scenario. This allows students to take risks and challenge themselves while feeling supported [4].

To address this concern, the project "Simulated Placement and Rounds" (SPaR) was created. Focusing on these practical skills in a simulated environment, it aimed to ease the transition from final-year medical students to foundation doctors. The project measured perceived confidence of students before and after the simulation rather than objective competence in carrying out a medical ward round. It focused on three key domains: preparedness for ward rounds, documentation, and managing an unstable patient.

Literature review

Similar studies identified educational interventions that used a simulation-based approach to improve ward rounds for medical students and resident doctors. Powell et al. examined a combination of classroom and group simulation to improve ward round practice and incorporate a ward safety checklist within their hospital trust [5]. This study used similar data-collection methods through pre- and post-training questionnaires that rated confidence in the skills taught. This appears to be an appropriate method of collecting the relevant data and allows for effective comparison between the two data sets. The simulation-based element of the training took place in a group setting, where only a limited number of students completed the simulation and the rest observed, with delegated roles during the debrief. This was an effective way to engage large numbers of students in learning but limited the simulation experience for individuals. Furthermore, this study included both early-year foundation doctors and medical students, recognizing that this skill is not adequately covered in the usual medical school curricula.

A project by Morgan et al. [6] used a low-fidelity simulation to emulate a ward round, focusing on clinical tasks such as discharge summaries, prescribing, and handovers. It was also designed for undergraduate medical students to help ease the transition to foundation practice and build confidence in ward round skills. This study used a similar data collection approach, measuring pre- and post-confidence scores with a Likert scale. Additionally, this study collected preliminary data from foundation doctors on perceived preparedness and confidence for practice; this provided a basis for developing the project and its specific goals. It had a larger sample size of 133 medical students, especially compared with the SPaR project, and was based in a single UK medical school. The results demonstrate significant improvement in confidence around the specific domains following the simulation course. This study focused less on students leading the ward round, which the article also acknowledged, and a limitation was the simulation's low fidelity. However, this may make it easier to apply more broadly, as it reduces the need for high-fidelity resources.

While reviewing the existing literature, it was noted that other interventions also involve augmented reality and simulation to teach medical ward rounds. Rainforth et al. used a quasi-experimental mixed-methods design to investigate student confidence in ward rounds [7]. It used a similar approach, collecting quantitative data through pre- and post-questionnaires and qualitative data through focus groups. The quantitative questionnaires asked students to rate their confidence, a measure of success also used in the present project. Notably, this was a small study, and the intervention may be less generalizable to wider settings because augmented reality is less available than simulation or simulated patients. Overall, the study showed possible positive impacts of augmented reality, but the data were inconclusive when comparing it with simulation; therefore, it could not determine whether one method is better than the other. This innovative method could be further explored in another educational intervention.

Le et al. conducted a systematic review of both the positive and negative impacts of simulated ward rounds [8]. Some positive impacts highlighted include the ability to challenge, troubleshoot, and learn how to improve in a safe environment. The SPaR project incorporated these principles. Negative elements of these interventions demonstrate the complexity and cost of facilitating these simulations. A limitation of the studies in this systematic review was the lack of follow-up on how transferable and applicable the skills were going forward. This is an interesting point to consider for future simulation-based or skill-based interventions. This review covered studies that have incorporated ward round simulations for medical students. Systematic reviews can include a wide range of studies and sit high in the evidence hierarchy. Because educational initiatives are flexible and reflective, studies are difficult to compare reliably, as they often combine qualitative and quantitative data. A meta-analysis was unable to be carried out in this study.

The literature review also identified studies on students’ emotions and cognitive load in ward-round-based simulations. This allowed exploration of other aspects of ward round-based simulation beyond its impact on students' confidence. Both studies by Behrens et al. [9,10] used an emergency-based ward round as the foundation. One examined students' cognitive load during these simulation-based interventions and concluded that ward round simulations can improve clinical ward skills because extraneous cognitive load is not high enough to impair learning. The authors recommend ward-round-based simulation within the medical school curriculum [9]. Another study [10] explored academic emotions during simulation and found that students can experience positive emotions like pride and moderate anxiety, which can drive learning. Still, they can also experience emotions such as shame, which can be detrimental to students. The authors recommend designing simulation scenarios with student emotions in mind to optimize learning. Both studies used mixed-methods approaches that collected quantitative and qualitative data: subjective data from focus groups or interviews and objective data from performance assessments using a specific scoring tool. This variety of data can make comparisons to other studies difficult, especially when analyzing qualitative or self-reported data. These studies were based in a single center in Chile, which makes them less applicable to the SPaR study given the location and differences in common medical curricula.

While many studies investigate the role of simulation or augmented reality in preparing for ward rounds, study designs and outcomes vary widely. Despite this, a recurring theme is that, regardless of method, participants feel ill-prepared to conduct a ward round and much more prepared after the educational intervention. Evidence also encourages consideration of student cognitive load and emotions when developing these simulations. The SPaR project aims to add to this growing body of evidence supporting experiential learning in bridging the gap between theoretical knowledge and practical skills.

## Materials and methods

Program structure

This project was undertaken within a local NHS district general hospital and involved final-year undergraduate medical students during their medicine placement. These students were all from the same UK-based university. Due to non-attendance, 18 of the 21 invited students took part in the simulation over six months.

Key skills for a foundation doctor were identified as areas of focus, including documentation, task prioritization, clinical reviews, patient communication, and prescribing. Using these and common ward clinical scenarios, we designed a three-station simulated ward round. One station allowed students to review a patient independently; the patient required drug counseling and a next-of-kin update. This station focused on ward round clinical review, prescribing common medications, using guidelines, and communicating with both patients and family members. The following station involved a consultant-led ward round. The student was expected to document appropriately, prescribe medications, and communicate with team members. The station aimed to increase students’ confidence in clarifying and speaking up within a team, especially with senior clinicians when needed for patient safety. The final station began with the student writing a discharge summary, then a nurse asked them to review an unstable patient. This station was completed in a high-fidelity simulation suite and aimed to improve task prioritization and assess and manage an unstable patient using the A-E framework.

Students received a brief before each station and were given five minutes to read it and prepare. Students then had 25 minutes to complete each station. They received up to 10 minutes of individualized feedback from the facilitator on their performance, areas for improvement, and discussion of the station skills. Volunteer simulated patients also gave feedback on communication from the patient’s perspective. Students and facilitators met at the start and end of the session to set expectations, collect feedback, and address concerns in a safe, open environment. Care was taken to reduce potential emotional distress for students, and they had many opportunities to speak up if issues arose.

Data collection

Data was collected through pre- and post-simulation questionnaires. This involved collecting anonymized demographic data on the students, along with qualitative and quantitative data about the experience. Students were informed how their data would be used and were invited not to complete the forms if they did not want to participate, with no consequences to their session. All students (n = 18) participated in the study and consented to provide data.

A Likert scale was used to collect numerical data on students’ confidence in preparing for and participating in a medical ward round, documenting in a clinical environment, and independently managing an unstable patient. The scale ranged from 1 (not confident at all) to 10 (extremely confident). The questions were repeated after the simulation so scores could be compared.

At the end of the questionnaire, students answered two short-answer questions about what they felt was positive about the simulation and how it could be improved. This collected qualitative data regarding the simulation. All students answered all questions, resulting in 18 fully completed questionnaires for analysis.

Data analysis

Quantitative Likert-scale data were analyzed to identify differences between pre- and post-session questionnaires in the areas discussed. The data were analyzed using the Wilcoxon signed-rank test, the most appropriate analysis for small sample sizes and paired ordinal data. This test is appropriate for non-normally distributed data, as assumed in this study, and serves as a non-parametric alternative to the paired t-test. This allowed a clear comparison of scores and demonstrates the simulation's self-reported effectiveness in these areas. Effect size was calculated using a standardized Z-based effect size ($r = \frac{Z}{\sqrt{N}}$) to provide further evidence of change in confidence.

Due to an error during the development of the original questionnaires, we measured confidence in participating in a ward round in the pre-session questionnaire but not in the post-session questionnaire. This information is demonstrated in the appendices. To maintain consistency in data collection, we used the original questionnaire for all participants; therefore, this domain is not reflected in the results, as we could not compare pre- and post-session confidence levels. The preparedness domain shown in the results maps directly to the question on preparing for a ward round in both the pre- and post-session questionnaires. The other two domains also have dedicated questions within the questionnaires.

The qualitative data from the short-answer questionnaire were analyzed using thematic analysis. This allowed common themes to emerge from the answers, highlighting areas for improvement as the simulation continued. The thematic analysis followed Braun and Clarke’s six-step approach [11]. Data were collated into a table from individual questionnaires. Both authors generated initial codes using a theoretical approach and identified themes at a semantic level. Both authors reviewed, refined, and named themes, reaching consensus on disagreements through discussion. The results section discusses the main themes.

Ethical statement

According to the UK Health Research Authority decision tool, this project did not constitute research and therefore did not require NHS REC approval. It was conducted as a service evaluation of an educational intervention. All students gave voluntary informed consent to participate, and we anonymized the data.

## Results

Pre- and post-session questionnaires were collected from each participant to establish a baseline measure and evaluate the intervention's effectiveness. Responses prior to the session indicated moderate self-reported preparedness for ward rounds and documentation tasks, with notably lower confidence in managing an unstable patient. In the initial background questions, all students (n = 18) reported previous ward round experience, providing context for their confidence before the session.

Table 1 shows that, following participation in the simulation, confidence and self-reported preparedness improved across all assessed domains. Mean preparedness for ward rounds increased from 5.06 (SD = 1.54) pre-session to 8.28 (SD = 0.93) post-session. Confidence in clinical documentation improved from 5.33 (SD = 1.37) to 8.28 (SD = 1.15), while confidence in managing an unstable patient increased from 3.39 (SD = 1.01) to 6.44 (SD = 1.26). As pre- and post-session scores were obtained from the same participants, the Wilcoxon signed-rank test was used to assess changes in confidence across the three domains. Statistically significant improvements were demonstrated in preparedness (Z = -3.73, p < 0.001, r = 0.88), documentation (Z = -3.75, p < 0.001, r = 0.88), and managing an unstable patient (Z = -3.67, p < 0.001, r = 0.89). Effect sizes (r) across all three domains were large for this sample size and showed substantial improvement in confidence following the SPaR simulation. Figure 1 compares the mean pre- and post-session confidence scores.

**Table 1:** Pre- and post-session questionnaire scores comparing self-reported confidence across domains (n = 18) Data are presented as mean ± SD. Paired pre- and post-session scores were compared using the Wilcoxon signed-rank test. n represents the total number of participants with complete paired data. Zero-difference pairs (i.e., participants with identical pre- and post-session scores) were retained in the dataset and descriptive analyses but excluded from the Wilcoxon signed-rank calculation, as they contribute no rank; therefore, the analysis of managing an unstable patient was based on 17 non-zero pairs despite n = 18 participants. A two-sided p < 0.05 was considered statistically significant. W: Wilcoxon signed-rank test statistic, Z: standardized test statistic, p: p-value, r: absolute effect size calculated as $\frac{|Z|}{\sqrt{n}}$, SD: standard deviation

Domain	Pre-session mean ± SD	Post-session mean ± SD	n	W	Z	p-value	Absolute value of effect size (r)
Preparedness	5.06 ± 1.54	8.28 ± 0.93	18	0	-3.73	<0.001	0.88
Documentation	5.33 ± 1.37	8.28 ± 1.15	18	0	-3.75	<0.001	0.88
Managing an unstable patient	3.39 ± 1.01	6.44 ± 1.26	18	0	-3.67	<0.001	0.89

**Figure 1:**
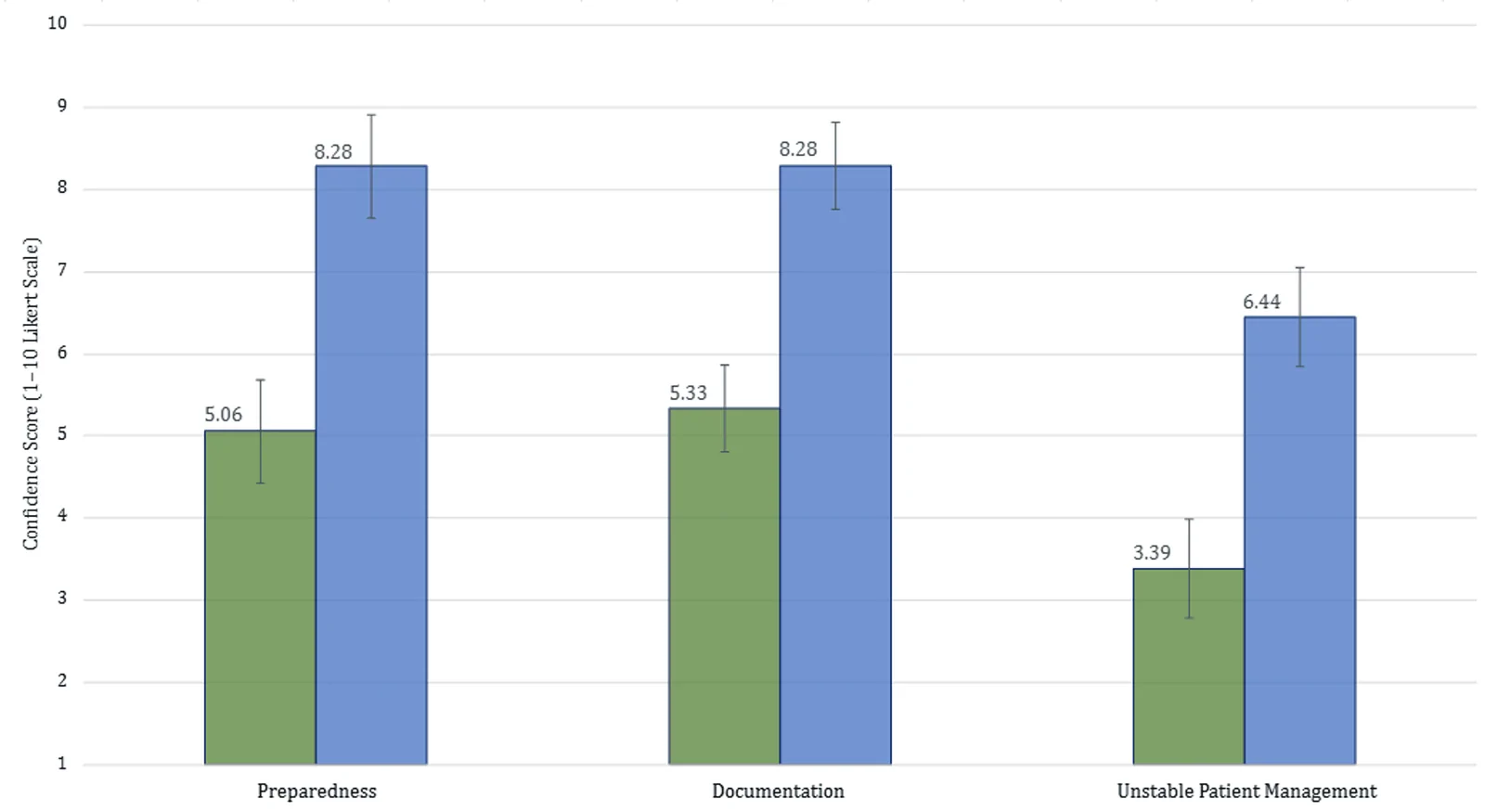
Grouped bar chart comparing pre- and post-session scores across measured domains (n = 18) The x-axis shows the three domains assessed, and the y-axis shows mean confidence scores measured on a 10-point Likert scale. Green bars indicate pre-session confidence scores, and blue bars indicate post-session confidence scores. Error bars represent the 95% CIs. Numeric values are displayed above each bar. CIs: confidence intervals

Post-session ratings indicated high perceived usefulness of the simulation, with consistently strong scores for overall value and debriefing effectiveness. Thematic analysis identified several key intervention strengths. Participants frequently highlighted the realism of the scenarios and opportunities for hands-on prescribing and practice in task prioritization. Participants consistently praised the quality of feedback and debriefing, describing it as thorough, constructive, and helpful in consolidating learning. Students also described increased confidence in applying A-E assessment frameworks, communicating with patients and next of kin, and documenting consultant-led ward rounds.

Suggested areas for improvement formed additional themes. These primarily included a desire for more and broader scenarios, clearer pre-briefing before simulation stations, and more opportunities to practice discharge summaries and take-home prescriptions. Table 2 presents some of the highlighted themes, with direct quotes from students that illustrate them.

**Table 2:** Qualitative themes and example quotations This table presents the overall themes identified through thematic analysis of the qualitative data, along with brief example quotations from students that illustrate these themes.

Theme	Example quotations
Scenario realism	"The reality of the situations was great", "Real life situations"
Hands-on prescribing	"Really useful to practice prescribing", "I feel more confident prescribing", "More practice prescribing and TTOs"
Task prioritisation	"Learnt how to prioritise"
Quality feedback	"Really useful feedback", "Thorough reassurance and feedback", "Excellent feedback that was really productive and constructive"
Greater number and breadth of scenarios	"More stations", "More scenarios", "More different cases that we could encounter on the wards"
Clearer pre-briefing	"More clear briefs before starting", "More brief before going into sim"
Discharge summary practice	"Practice writing a discharge summary", "Proforma for discharge summary", "How to write a discharge summary"

Overall, the themes identified through qualitative analysis aligned closely with the quantitative findings, supporting the conclusion that the simulation was perceived as a realistic and effective preparation for foundation-level ward round practice. The study also identified actionable improvements for future iterations.

## Discussion

Educational impact of experiential learning

This program demonstrated a statistically significant improvement in self-reported confidence scores across all assessed domains, including preparedness for ward rounds, documentation, and managing an unstable patient. Notably, confidence in managing an unstable patient showed one of the largest mean increases, rising substantially from the lowest pre-intervention score. This suggests that simulation may be particularly useful in addressing low perceived confidence in managing unstable patients among final-year students [3]. Simulation-based teaching in the literature has been associated with improved clinical performance, including reduced error rates and improved quality of care delivered [3]. Although the SPaR project did not measure it, learners who participated in simulation have also demonstrated improvements in diagnostic accuracy and more timely evaluation and management of patient presentations [3].

For simulation to be effective, the learning environment must promote psychological safety. Learners should feel appropriately challenged within their level of competence while also being supported to make mistakes without fear of consequences. Structured and thorough debriefing, as incorporated into this program, is central to this process. Debriefing facilitates reflective learning, reinforces key clinical principles, and provides constructive feedback, thereby maximizing the educational impact of the simulation experience [3].

SPaR facilitators were tasked with intentionally embedding Kolb’s theory of experiential learning into their debriefing approach [12]. This ensured that learning extended beyond simple feedback and instead facilitated deeper cognitive processing and behavior change [13]. Facilitators responsible for debriefing had received prior training in medical education and feedback delivery, helping ensure a consistent, structured approach in a psychologically safe learning environment. Features such as the "Diamond Debrief" model, as described by Jaye et al., provided scaffolding for a structured, constructive debrief [14]. The principles of this method describe the ideal process, consisting of ’opening out a facilitated discussion about the scenario before bringing the learning back into sharp focus with specific learning points’ [14]. Faculty training supported standardization across sessions and minimized variability in teaching, reinforcing a high-quality educational experience aligned with the program’s learning objectives.

Kirkpatrick model alignment

The SPaR program aimed to follow the Kirkpatrick Model, which assesses training outcomes across four levels [15]. At Level 1 (reaction), collected feedback indicated high engagement and satisfaction with the session. The SPaR project also captured self-reported improvements in knowledge, skills, and confidence in clinical and communication tasks.

Because the study used self-reported outcomes, it could not objectively demonstrate level 2 (learning), as it included no objective measures of learning and performance [15]. Because the project focused on students' learning and development, measuring objective performance could have changed the experience; therefore, the study used self-reported outcomes. To better align with the Kirkpatrick model and capture learning, future iterations could include some objective outcome measures. The study did not directly observe Level 3 (behavior) or Level 4 (results) during these sessions. However, learners reported increased confidence and preparedness for real-world ward round tasks, suggesting potential for transfer of skills to clinical practice. This is particularly relevant in medical education, as increased confidence and preparedness in final-year students have been linked to smoother transitions into foundation doctor roles [2]. By demonstrating measurable gains in reaction and self-reported outcomes, and by providing indicators of potential behavioral transfer, the SPaR program supports evidence-based approaches to bridging the gap between undergraduate education and safe, competent clinical practice.

Embedding constructivist principles

Furthermore, SPaR is grounded in constructivist pedagogy. Jean Piaget’s constructivist approach suggests that learners build their own understanding through active participation rather than passively receiving information [16]. Traditionally, medical student placements can be quite passive, with students often observing from the back of the ward round and having limited opportunity to engage or make decisions [17]. In contrast, in the SPaR simulation, students took active roles and had to make real-time decisions, such as when to speak up, when to escalate concerns, and how to communicate with patients and family members. This shift from passive observation to active participation encouraged learners to build their own knowledge and clinical approach, making it more meaningful and transferable to real practice [16].

From a cognitive constructivist perspective, this process also supports the development and refinement of schemas, mental frameworks that help organize and interpret clinical information [16]. Through repeated exposure to realistic scenarios and reflection, students can adapt existing schemas or develop new ones, allowing them to approach similar situations more effectively in the future. This not only promotes deeper learning but also makes it more likely that the skills and behaviors developed will transfer into real clinical practice.

Challenges and limitations

While the program yielded positive outcomes, it also had challenges and limitations. Logistical constraints, including faculty availability, competing student timetables, and restricted access to the simulation suite, limited both the frequency and duration of sessions. With increased access to these resources, the program could potentially reach more students across a wider range of specialties.

Although participant feedback indicated a desire for additional stations, implementing this would necessitate further trained personnel and increased faculty availability. Expanding the number of stations may also risk exceeding students’ cognitive capacity, potentially reducing opportunities for meaningful reflection and diminishing key learning takeaways.

The program evaluation relied on self-reported measures rather than objective assessments. This allowed for a more open and flexible learning environment, which was the program's priority. However, this meant improvements reflected perceived preparedness rather than objectively demonstrated competence. Furthermore, this data collection method introduces bias, such as social desirability, as students may have reported positive improvements to fit in with peers and the environment. Answers may also be influenced by demand characteristics, with students providing socially desirable responses to portray the project favorably and make it appear to have a beneficial impact, especially since participants knew the project authors. Response shift should also be acknowledged, as the data were collected immediately after the simulation and no longitudinal data were collected, as mentioned in other limitations. Students' confidence could change over time and with experience. Potential biases in the project should be recognized as a possible limitation; however, they are difficult to reduce with the methodology used. Longitudinal data collection could help address response shift, and data collection by independent researchers may reduce investigator influence on participants’ responses.

Finally, the study was conducted within a single center with a small sample size, which may limit generalisability. Because all students were from the same cohort, a single medical school, and had previous ward round experience, as reflected in the baseline characteristics, the project may be limited by selection bias. Therefore, it does not represent all undergraduate medical students. Additionally, outcomes were measured immediately after the session, so the study did not assess longer-term retention or transfer to clinical practice. This limitation is reflected in similar studies within the systematic review by Le et al. [8].

Implications for curriculum design

Alongside the logistical constraints previously identified, the program was notably resource-intensive. Each simulation session, accommodating three students, required at least three facilitators trained in structured feedback delivery, plus up to four supporting faculty members to serve as patients, family members, nurses, and simulation technicians. In many educational contexts, recruiting, training, and maintaining these staffing levels regularly can be challenging, particularly when clinical or academic commitments limit faculty time.

From a curriculum design perspective, this highlights key considerations regarding scalability and sustainability. While low facilitator-to-student ratios can improve feedback quality and deepen learning, organizational and financial constraints limit them. Institutions aiming to integrate this program into a longitudinal curriculum would need to carefully balance educational benefits with practical feasibility. Investing in prior training in teaching and facilitation methods may help optimize staff effectiveness and support the program’s long-term success.

Future development

Further research incorporating objective, performance-based assessments and longitudinal follow-up would be valuable. This would help determine whether improvements in self-reported confidence and skills are maintained over time and whether they translate into sustained clinical competence and safe practice during the transition to foundation training. Such research could provide stronger evidence for the long-term impact of simulation-based ward round programs on trainee preparedness and patient outcomes.

## Conclusions

The SPaR simulation program demonstrated measurable improvements in final-year students’ confidence and self-reported preparedness for ward round skills. Using the Wilcoxon signed-rank test and effect size calculations, quantitative feedback showed statistically significant gains across all measured domains. Qualitative feedback further supported these findings, with participants rating the sessions highly for engagement, realism, and feedback quality. SPaR incorporated Kolb’s experiential learning theory and followed the Kirkpatrick model to reinforce key principles in medical education. Although the study did not assess patient-level outcomes or long-term skill retention, the findings suggest that simulation-based training may help bridge the gap between undergraduate education and safe, competent foundation practice. Future studies incorporating objective performance measures and longitudinal follow-up are warranted to confirm the lasting impact of such interventions on clinical capability and patient care.

## Appendices

Simulated placement and rounds (SPaR): student feedback form

**Table 3:** Demographic information

Variable	Response
Year of study	
Medical school	
Previous ward round experience (yes/no)	
Gender	
Age	

**Table 4:** Pre-session confidence assessment

Question	Response scale (1–10) 1 = not confident at all; 10 = extremely confident
Confidence in preparing for a ward round	
Confidence in documenting in patient notes during a ward round	
Confidence in managing an unstable patient independently (assessment, management plan, documentation)	
Confidence/preparedness to participate in a clinical ward round at this stage	

**Table 5:** Post-session feedback assessment

Question	Response scale (1-10) 1 = not useful; 10 = extremely useful
Usefulness of the simulated ward round overall	
Confidence in preparing for a ward round following the session	
Confidence in documenting in patient notes during a ward round following the session	
Confidence in managing an unstable patient independently (assessment, management plan, documentation) following the session	
Effectiveness of the feedback/debrief session	

**Table 6:** Qualitative feedback

Question	Response
Please list up to three things that went well in this session	
Please list up to three things that could be improved for next time	

**Table 7:** Consent statement

By completing this form, you are providing your consent to participate in this project. All data collected will be anonymized to protect your privacy and confidentiality. Participation is voluntary, and you may choose not to complete the form if you do not wish to take part. The anonymized data may be analyzed and disseminated, including possible publication in a peer-reviewed medical education journal, for the purposes of advancing knowledge and practice in the field. You may withdraw consent at any time prior to publication.
Please sign here
